# Morphologically Conservative but Physiologically Diverse: The Mode of Stasis in Anostraca (Crustacea: Branchiopoda)

**DOI:** 10.1007/s11692-014-9283-6

**Published:** 2014-05-25

**Authors:** Markus Lindholm

**Affiliations:** Norwegian Institute for Water Research/NIVA, Gaustadalleen 23, 0349 Oslo, Norway

**Keywords:** Living fossils, Stasis, Pond, Anostraca, Branchinecta

## Abstract

The essay discusses whether biotic and abiotic environments differ in their ability to speed up or slow down morphological change and the generation of new lineages. Examples from the class Branchiopoda show that morphological conservatism is associated with enemy free space in species-poor habitats dominated by abiotic factors, while Red Queen mechanisms are predominant in larger systems with complex biotic interactions. Splitting of Branchiopod main lineages is associated with increased fish predation during the Devonian. The order Cladocera adapted and remained in larger aquatic systems, and subsequently generated a variety of new families, genera and species. The order Anostraca, on the other hand, maintained its ancestral morphology and survived only as “living fossils” in isolated ponds of harsh habitats. Despite their archaic morphology, however, they possess highly sophisticated adaptations to local physicochemical properties of their extreme environment. Hence, although morphologically conservative and possessing traits typical for “living fossils”, anostracan physiological abilities are closely adapted to the challenging and variable physicochemical conditions of ponds and ephemeral pools.

## Introduction

Biological novelties become part of a population by means of natural selection, because factors in the environment that affect survival and reproduction favor specific adaptations. Many of these factors are themselves biological, in terms of parasites, symbionts, predators, prey or competitors. But even physicochemical conditions—UV radiation, temperature, drought, pH, salinity or mechanical stress—evoke biological novelties. However, the evolutionary effects of abiotic factors on the one hand, and biological factors, on the other, may differ. Adaptive responses to the living environment tend to generate biological feedbacks, leading to continued fitness shifts in competing and mutually adapting lineages (Van Valen 1973; Stenseth and Maynard Smith 1984; Price 1988; Liow et al. 2011; Lawrence et al. 2012). The fine-tuned adjustments of inner organs in the animal body illustrate the principle, but it can be recognized wherever biotic factors have dominated the environment over geological time, such as rain forests or coral reefs. In fact, it has been claimed that most terrestrial novelties since the Cambrian arose in biotic, co-evolving environments (Price 2002; Benton 2009).

Similar dynamic feedback loops are less accentuated where adaptations relate to physical factors. Melanin does not alter the UV radiation regime, and thickening of leaf cuticle does not affect the tropical rain. Physical factors remain mainly unaffected by biological adaptations, making further adjustments less urgent (Butterfield 2007). Hence, while adaptations to biological agents possibly drive populations into nearly never-ending Red Queen dynamics, the corresponding morphological responses to physical factors are rather stagnant. Such differences could lead to different evolutionary histories and shed light on possible causes for the apparent imbalance between periods of morphological stasis and episodes of rapid lineage splitting (Eldredge et al. 2005; Hunt 2007).

This essay discusses if abiotic factors can help to explain morphological conservatism in certain crustaceans. Comparing two taxa of freshwater branchiopods, it explores how biological or physicochemical factors over geological time have driven lineages in different directions.

## The Origin of Branchiopods

**Fig. 1:**
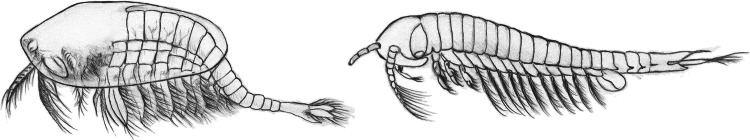
Two primitive branchiopods, the Cambrian *Rehbachiella kinnekullensis* from Sweden (*left*), and *Lepidocaris rhyniensis* from the Devonian in Scotland (*right*) (drawings by K. Skilbrei)

The class Branchiopoda comprises small to medium sized (0.2–100 mm) crustacean, primarily inhabiting freshwater. The taxon is rather small, amounting to 1,200 species worldwide, and molecular studies have settled their monophyletic origin (Richter et al. 2007). The class comprises ten orders, with Anostraca (fairy shrimps) and Cladocera (water fleas) as the most familiar. Their fossil record reaches back to the Paleozoic (Walossek 1998; Boxshall 2004), where the Anostraca is considered as the most ancient (Schram and Hof 1998). Their scattered, but worldwide distribution also points to a pre-Gondwanian origin. The oldest known anostracan fossil is *Rehbachiella kinnekullensis* from Swedish Cambrian rocks (Fig. 1, left). Their well preserved, leaf-like filtering appendages closely resemble those of recent descendants. Moreover, Harvey et al. (2012) reported well-preserved mandibles and filtering appendages of Cambrian branchiopods from Canada, nearly identical to those of recent anostracans. Another early species is the Devonian *Lepidocaris rhyniensis* from Scotland. Its segmented body and the lack of carapace are primitive characteristics even among recent fairy shrimps, and present juvenile stages closely resemble those of *Lepidocaris*, as well (Fig. 1, right). Altogether, these records suggest only weak morphological changes, and point to morphological conservatism as a main characteristic in the order Anostraca. Why was this ancient morphology maintained?

## The Community-Structuring Effects of Fish Predation

A fateful event in the branchiopod history was the dawn of predatory fish, which accelerated during the Devonian, following fish novelties, such as fins, jaws and teeth (Wägele 1992; Rücklin et al. 2012). Aquatic invertebrates found themselves exposed to new and far more intense predation regimes. Cladoceran characteristics departed and diversified during the following period (Womack et al. 2012), in terms of life history, reproductive mode, body size and morphology. Firstly, they became so small that most fish fed on them only during the fry phase. The characteristic reproductive mode of cyclic parthenogenesis, moreover—a characteristic for many microcrustaceans—allows high growth rates even when facing considerable predation pressures (Schmit et al. 2012). Recent forms also avoid visual predation by migration into deeper water layers at daytime, by increased transparency, or by formation of spines which make them less edible (Lindholm 2002; Laforsch and Tollrian 2004; Johnson et al. 2006). Their ability to co-exist with their principal predators allowed for various secondary adaptations, which made cladocerans the most diverse and successful taxon of all branchiopods (Adamovicz et al. 2009). The genus *Daphnia* evolved a suite of features associated with pelagic life, such as vertical tilting of the body axis and a pelagic driven, niche-partitioned and size-specific phytoplankton diet. Another successful lineage (Chydoridae) adapted to littoral microhabitats, or to floating particles on the water surface (*Scapholeberis* sp.). Some cladocerans returned to marine habitats (*Evadne* sp.), while others (*Bythotrepes* sp., *Leptodora* sp.) became predatory. Cladoceran ability to co-exist with fish hence opened up opportunities for numerous morphological novelties.

**Fig. 2:**
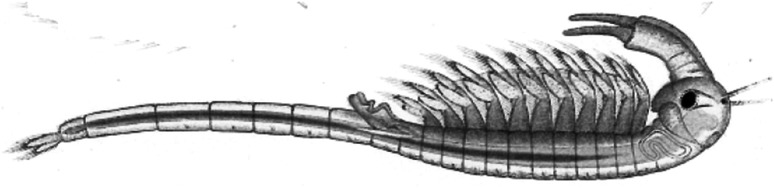
The fairy shrimp *Branchinecta paludosa* (drawing by G.O. Sars)

Anostracans responded different. Despite considerable size, they retained their archaic morphology. They still move slowly upside down through the open pelagial, filtering particles from the water, unable to hide in the bottom substrate, and hence an easy prey for fish (Fig. 2). The extreme rarity of coexistence between fairy shrimps and fish is consistent with this pattern, as are the detrimental effects of fish stocking on fairy shrimp populations (Eriksen and Belk 1999; Lindholm et al. 2012). Anostracan only survived in small, isolated habitats with peculiar features, and devoid of fish: shallow ponds and ephemeral pools, predominantly in inhospitable and extreme environments.

## Ponds and Ephemeral Pools are Harsh Habitats

Ponds and pools possess features which in many respects differ profoundly from larger water bodies. Thermal structuring shelters most perennial lakes from meteorological extremes and facilitates stable conditions for temperature, oxygen, pH and nutrients. The biota is accordingly protected from sudden, steep physicochemical shifts (Jackson et al. 2001; Wetzel 2001). The stability contributes to niche separation and co-existence, and hydrological connectivity between lakes facilitates migration and higher biodiversity, as well (Oberdorff et al. 1997).

Ponds and ephemeral pools—rainwater filled depressions of warm-arid deserts, prairie potholes, volcanic mud-flow pools, melt-water ponds in alpine or arctic environments or vernal pools in winter rain climates—are different. Most attempts to typify such water bodies have been based on physicochemical properties. Wiggins et al. (1980) emphasized the lack of hydrological connectivity between ponds and the dependence of true pond dwellers on dry resistant resting eggs to bridge desiccation periods. Williams (1985), working in semiarid environments, considered the degree of hydrological predictability and salinity as particularly important. Eng et al. (1990) classified three main groups of ponds, according to somewhat different variables: Episodic pools which fill and dry up repeatedly, perennial ponds, which exhibit large seasonal water level oscillations but rarely dry up, and aestival ponds, which are permanent, but shallow and freeze to the bottom during cold seasons. The latter classification circumscribes pond types inhabited by fairy shrimps.

The typology hides large local differences, though, and most ponds go through considerable seasonal shifts in physicochemical frame parameters (Eriksen and Belk 1999). Some become extremely clayish or saline, which cause strong fine-scaled thermal stratification (Eng et al. 1990). Others are clear and oscillate closely with atmospheric temperatures. Their unpredictable nature and rapid changes make aquatic vegetation poor, and size limits habitat diversity, as well. Most are rainwater fed (Williams 1985; Baskin 1994) or snowmelt driven (Lindholm et al. 2012) and, depending on local bedrock, accordingly prone to acidification. Some develop peculiar water chemistry, as in soda pans and seasonal salt lakes (Eng et al. 1990; Brandan et al. 1993). Extreme salinity, alkalinity, turbidity and pH are common in warm-arid regions (Gonzalez et al. 1996; Hebert et al. 2002), as is strong UV radiation (Sommaruga 2001; Marinone et al. 2006; Rautio et al. 2009) and rapid thermal oscillations in alpine and polar clear water pools (Peck 2005; van der Wal and Hessen 2009; Hegna and Lazo-Wasem 2010). Altogether, ephemeral ponds and pools belong to the most inhospitable and unpredictable habitats the earth, and, as might be expected, comprise impoverished diversity and comparatively poor biotic interactions (De Meester et al. 2005; Ripley and Simovich 2009). Some insects, such as Dytiscides and Notonectids, may exploit resources of ephemeral pools. A number of crustacean, notably ostracods, cladoceran and copepods, inhabit pools and ponds, as well. But fairy shrimps have limited their occurrence entirely to these extreme habitats.

## Morphologically Conservative but Physiologically Diverse

The emerging pattern is hence evolutionary lineages rather exposed to physicochemical than to biotic stressors. Sheltered from competition and predation, fairy shrimps managed to maintain their archaic morphology and behavior. Instead of getting entangled in Red Queen dynamics, they ended up as “fugitive species” (Hutchinson 1951) in largely enemy-free habitats, devoid not only of fish, but also of other potential competitors or invertebrate predators (Hurlbert et al. 1986). The pools and ponds instead demanded peculiar physiological abilities, in order to cope with the rapid local changes in physicochemical frames, or with permanent extreme conditions.

A closer look at the large and widespread anostracan genus *Branchinecta* may illustrate this point. In arid California, *B. lindahli* starts its life in rainwater pools at temperatures close to zero, which however approaches 35 °C during summer (Eriksen and Belk 1999). In Antarctic melt water pools, *B. gaini* is exposed to diurnal temperature oscillations amounting to 25 °C (Peck 2005). Alpine ponds in higher latitudes of Europe, where *B. paludosa* hatches at snow melt, also regularly reach 25 °C after few weeks (pers.obs.). Until recently were the plentiful vernal pools in the southeastern European steppe region inhabited by *B. ferox*, but these habitats are now mainly extinct. A number of species persist in seasonal ponds where salt levels exceed that of sea water during the last period before dry up (Alonso 1990; Brandan et al. 1993). Other Branchinectids are adapted to permanent extreme conditions. In the Andes, *B. brushi* inhabits clear-water pools close to 6,000 m.a.s.l., with strong UV loads (Hegna and Lazo-Wasem 2010). And chlorinated and sulphated ponds in the neighboring Atacama Desert are preferred habitats for *B. papillata* (Rogers et al. 2008). Other species, such as *B. lindahli,* has adapted to extreme alkaline water, with pH approaching 10 (Eng et al. 1990). Hence, although morphological conservative are fairy shrimps physiologically highly diverse, and in part also highly flexible in facing steep physicochemical shifts.

The combination of morphological conservatism and physiological flexibility found in many anostracan has much in common with the findings of Wake et al. (1983), who argued that morphological stasis in the salamander family Plethodontidae was intimately connected to their unusual flexible behavior. It also relates to Sheldon’s (1996) concept of stasis in rapid fluctuating environments. While environments dominated by biological interactions counteract physical fluctuations and promote niche separation and lineage splitting, could fluctuating physicochemical stressors favor flexibility, as “*selection soon tends to favor lineages with “all*-*purpose” hard*-*part morphologies that are relatively inert to each environmental twist and turn*” (Sheldon 1996, p. 213). Physiological generalists gain increased fitness in temporally heterogeneous habitats, which enable them to cope, and not to adapt, with periods of extremes. Relations between morphological conservatism and fluctuating environments are reported for other crustaceans, as well. A large scale example is the Pleistocene glaciation, which failed to cause enhanced speciation rates. Lineage conservatism during Pleistocene at northern latitudes is documented for a range of taxa, including vascular plants (Willis and Niklas 2004), insects (Coope 2004), marine invertebrates (Jackson and Sheldon 1994), birds (Zink et al. 2004) and mammals (Barnosky 2005).

Two very different realms—morphology and physiology—meet in every living being. The history of Branchiopods signals that the two not always go along identical evolutionary pathways. Following the advent of predatory fish of the Devonian, cladoceran adapted a broad suite of morphological and life history traits which allowed co-existence and complex Red Queen dynamics with competitors and predators. Anostracan, on the other, never solved the problem of coexistence with their principal predators, and found themselves marginalized to enemy free habitats in extreme environments, where they remained morphologically unaltered, but purified the adaptive interface between their physicochemical environment and their internal physiology.
